# Removal of Volatile Phenols From Wine Using Crosslinked Cyclodextrin Polymers

**DOI:** 10.3390/molecules25040910

**Published:** 2020-02-18

**Authors:** Chao Dang, Vladimir Jiranek, Dennis K. Taylor, Kerry L. Wilkinson

**Affiliations:** 1 The University of Adelaide, School of Agriculture, Food and Wine, PMB 1, Glen Osmond, SA 5064, Australia; chao.dang@adelaide.edu.au (C.D.); vladimir.jiranek@adelaide.edu.au (V.J.); dennis.taylor@adelaide.edu.au (D.K.T.); 2 The Australian Research Council Training Centre for Innovative Wine Production, PMB 1, Glen Osmond, SA 5064, Australia

**Keywords:** *Brettanomyces*, cyclodextrin, gas chromatography-mass spectrometry, nuclear magnetic resonance, smoke taint, wine

## Abstract

Volatile phenols have been implicated as contributors to off-odors associated with taints from bushfire smoke and microbial spoilage. Various methods for the amelioration of off-odors have been evaluated, but to date, they have not included cyclodextrin (CD) polymers. In the current study, two CD polymers were prepared from β- and γ-CD, using hexamethylene diisocyanate (HDI) as a crosslinking agent. Adsorption tests were performed with four volatile phenols (guaiacol, 4-methylguaiacol, 4-ethylguaiacol and 4-ethylphenol) at concentrations up to 1 mg/L. The removal of volatile phenols by CD polymers achieved equilibrium almost instantly, with isotherm tests suggesting an adsorption capacity of 20.7 µg of volatile phenol per gram of polymer. Langmuir and Freundlich models were subsequently used to fit the data. In batch adsorption tests, the CD polymers achieved 45 to 77% removal of volatile phenols. Polymer reusability was also evaluated and was found to be excellent. A comparison between volatile phenol adsorption by CDs vs. CD polymers, determined using a novel four-phase headspace solid-phase microextraction (HS-SPME) method for gas chromatography-mass spectrometry (GC-MS), suggests CD polymers offer several advantages for use by the wine industry.

## 1. Introduction

Aroma plays an important role in determining wine quality, so optimising the aroma profile of wine remains a key aim of the winemaking process. Despite the evolution of viticultural and enological techniques that facilitate this effort, challenges still exist for the wine industry, including off-odors that result from elevated concentrations of volatile phenols. In recent years, climate change has aggravated the risk of certain off-odors occurring in wine [1]. A known source of volatile phenols associated with off-odors is *Brettanomyces*/*Dekkera*, spoilage yeast that produces 4-ethylphenol and 4-ethylguaiacol, which impart animal, horse stable, sweaty and medicinal characters at excessive concentrations [2,3]. As a consequence of the warmer ripening conditions associated with climate change, grapes and therefore wine, tend to have less natural acidity (higher pH levels) and higher sugar concentrations, i.e., conditions which favor the growth of spoilage yeast [4]. The warmer, drier weather conditions also increase the risk of bushfires occurring near wine regions, which can lead to vineyard exposure to bushfire smoke, and another volatile phenol related off-odor, commonly known as smoke taint [5,6]. Elevated concentrations of guaiacol, 4-methylguaiacol, *o*-, *m*- and *p*-cresol, and syringol have been found in wines made from smoke-exposed grapes [7,8], along with objectionable smoke-related sensory characters that negatively affect wine quality [8,9]. 

Conventional techniques have been used to mitigate volatile phenol related off-odors in wine, including the use of sulfur dioxide to control the growth of *Brettanomyces* [10], and reducing the duration of skin contact during fermentation to minimise volatile phenol extraction from smoke-affected grapes [11]. In recent years, several studies have also evaluated the potential for volatile phenols to be removed from wine using activated carbon [12], polyvinylpolypyrrolidone [13], yeast lees [14], yeast cell wall [15] and cellulose [16] additions, reverse osmosis membrane filtration [17] or treatment with molecularly imprinted polymers [18]. However, these removal techniques inevitably impact the intensity of the desired aromas as well. In a recent study, cyclodextrins (CDs), a group of compounds that have long been used by other industries, were reported to be able to form inclusion complexes with volatile phenols in wine, reducing the volatility from these off-odor volatiles [19]. 

The most common naturally occurring CDs are α-CD, β-CD and γ-CD. These cyclic oligosaccharides consist of 6, 7 and 8 α-1,4-linked glucose subunits, respectively, and derive from starch [20]. The spatial arrangement of C-H bonds within each subunit of the ring-shaped CD molecule results in a lipophilic cavity (H-3 and H-5) with a hydrophilic surface (H-1, H-2 and H-4); a structure that enables CDs to form inclusion complexes with the non-polar moieties of guest molecules [21]. This binding process exists as a dynamic equilibrium in aqueous environments, and is mainly driven by non-covalent Van der Waals forces, resulting in a more stable status with lower energy when enthalpy-rich water molecules are replaced by non-polar guests [22]. The size, hydrophobicity, and conformation of the guest molecule influences encapsulation in aqueous conditions [23]. Formation of the CD-guest complex leads to a series of changes in both the physical and chemical properties of the guest molecule, including increased solubility (of insoluble compounds), protection from degradation, reduced volatility, and therefore, reduced aroma and flavor impact, as well as shifts in spectral peaks and chromatographic separation [20]. As a result of these functions, CDs have been exploited as additives by the food, beverage and flavor industries [22,24,25,26]. α-CD and β-CD are currently listed as novel foods in the US, the EU and Japan, whereas γ-CD is only approved in the US and Japan. α-CD and γ-CD are both listed as novel foods by Food Standards Australia–New Zealand, whereas β-CD is classified as a food processing aid. A recent report suggests there are more than 200 food products containing CDs as ingredients [27]. 

The complexation that occurs between CDs and various aroma volatiles has been widely studied, however, there is limited literature concerning the use of CDs in wine. A key barrier to the uptake of CDs for wine production is the strict regulation of permitted winemaking additives, which does not currently include CDs. There is, nevertheless, increasing interest in developing insoluble CD polymers to broaden the applications of CDs. CDs can be polymerised with various molecules, known as crosslinkers, with these compounds containing at least two functional groups that can react with the hydroxyl groups present on the glucose subunits of CD, thereby linking the molecules in a chain structure [28]. CD polymers have been used to remove phenols and dyes from wastewater [29,30,31,32], with several CD crosslinkers having been studied. Crini and colleagues crosslinked β-CD with epichlorohydrin, and studied the sorption capability of the resulting polymer with benzene derivatives, such as phenol, *p*-nitrophenol, and benzoic acid [29]. Yamasaki used hexamethylene diisocyanate (HDI) and toluene-2,6-diisocyanate as CD crosslinkers, and showed adsorption of cresols, phenol and xylenol from wastewater by the polymers [30]. Other studies have used chitosan and citric acid to form phenol adsorbing CD polymers for removing pollutants from water [31,32,33,34]. 

The present study aimed to evaluate the potential for CD polymers to remove four volatile phenols, guaiacol, 4-methylguaiacol, 4-ethylguaiacol and 4-ethylphenol (Figure S1), from tainted wine. Two insoluble CD polymers were prepared from β-CD and γ-CD using HDI as a crosslinker. Adsorption tests were subsequently conducted to evaluate the preference and capability of the polymers to adsorb volatile phenols associated with smoke taint and *Brettanomyces* spoilage. In order to compare the removal of volatile phenols following the addition of CDs and CD polymers, a newly developed four-phase headspace solid-phase microextraction (HS-SPME) method for gas chromatography–mass spectrometry (GC-MS) was employed to quantify changes in volatile phenol concentrations without interference between CD additives and internal standards [35]. 

## 2. Results and Discussion

### 2.1. Preparation of CD Polymers and Determination of Time Required to Achieve Equilibrium

**Figure 1:**
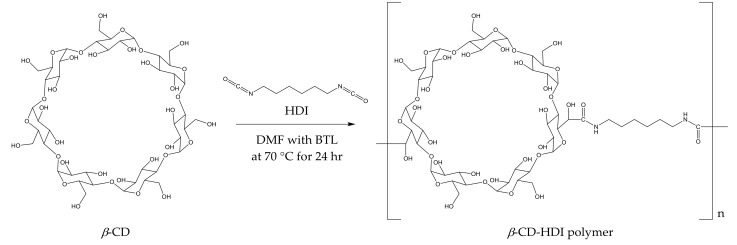
Preparation of β-cyclodextrin (CD)-hexamethylene diisocyanate (HDI) polymer and its hypothetical structure.

**Figure 2:**
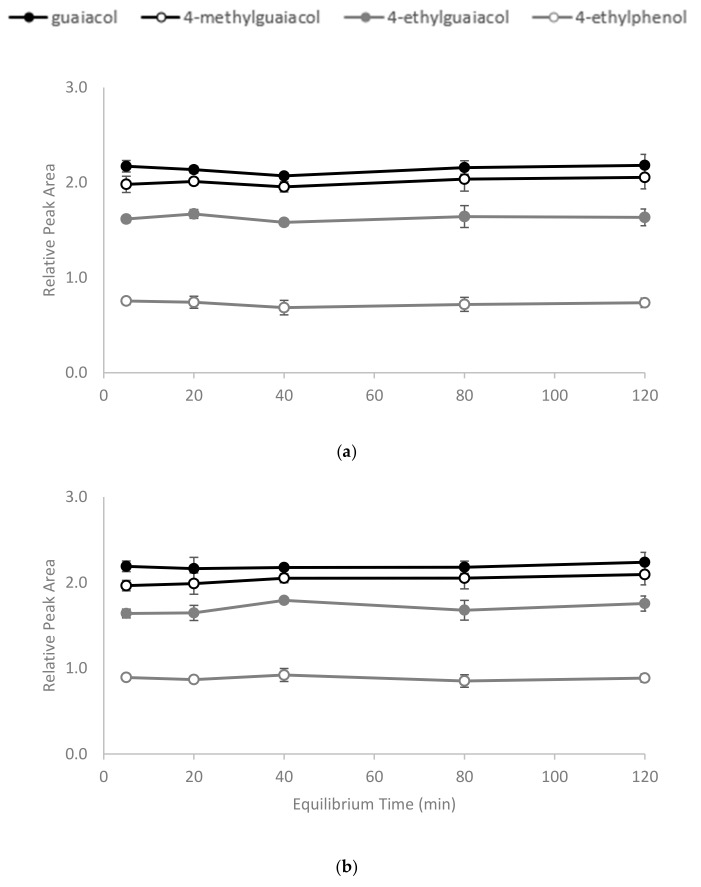
Equilibrium time for binding of volatile phenols by (**a**) β-CD-HDI and (**b**) γ-CD-HDI polymers, expressed as relative peak area. Values are means of three replicates ± standard error (but some standard errors are obscured by symbols). Values were not significantly different (one-way ANOVA, *p* = 0.05).

The polymerisation process (Figure 1) yielded a slightly yellow coloured polymer with β-CD and a white-coloured polymer with γ-CD. After ball mill grounding, the powdered polymers were weighed and added to wine samples. The time required for CD polymers to achieve adsorption equilibrium is dependent on the sample mixture, the chemical properties of the target molecule and temperature [30,31]. In the current study, 1% *w/v* of the polymer was added to spiked model wine samples at 25 °C, and an incubation time of less than 5 min was required for the adsorbents to achieve equilibrium. No significant differences in the relative peak areas (RPAs) for the four volatile phenols were observed between aliquots collected from the reaction mixture at 5, 20, 40, 80 and 120 min intervals after polymer treatments (Figure 2). The rapid equilibria observed were consistent with the findings of Yamasaki and colleagues, who used a β-CD-HDI polymer to remove phenols from wastewater [30]. Alsbaiee et al. used a different type of the β-CD polymer with tetrafluoroterephthalonitrile as their crosslinker, and also reported rapid equilibrium, i.e., within 10 min [32]. Other studies have reported different equilibrium times when using different CD polymers on various adsorbates. For example, Romo [33] and Crini [36] used epichlorohydrin crosslinked β-CD polymer to adsorb dyes and phenols, with 2 h of incubation required to achieve equilibrium. The differences in time to equilibrium may be attributed to the polymer type, the initial concentration of adsorbate and/or the sample matrix. 

### 2.2. Determination of CD Polymer Adsorption Capacity in Model Wine

**Figure 3:**
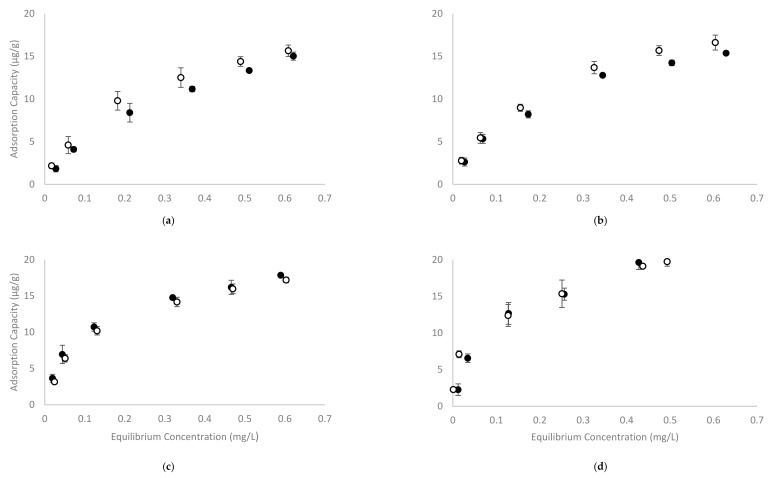
The adsorption capacity of (**a**) guaiacol, (**b**) 4-methylguaiacol, (**c**) 4-ethylguaiacol and (**d**) 4-ethylphenol by (●) β-CD-HDI and (○) γ-CD-HDI polymers in model wine at pH 3.5 and 25 °C. Values are means of three replicates ± standard error (but some standard errors are obscured by symbols).

The recently developed four-phase HS-SPME GC-MS method showed excellent repeatability for analysis of volatile phenols in model wine at concentrations ranging from 0.5 to 1.0 mg/L (using calibration curves spanning 0 to 2.0 mg/L, at 0.25 mg/L internals) [35]. To evaluate the adsorption capacity of CD polymers, 2% *w/v* of β-CD-HDI or γ-CD-HDI polymers were added to model wine samples containing sequential concentrations of volatile phenols (being the same concentrations used for the development of calibration curves). The RPA of treated samples represents the concentration of free volatile phenols, with the corresponding concentrations (C_e_) being calculated from calibration curves. The difference between C_0_ and C_e_ was considered to be due to volatile phenol adsorption by the polymer, and was used to calculate the adsorption capacity q_e_. Plots of q_e_ against the concentration of volatile phenols remaining once the adsorption equilibrium was achieved, are shown in Figure 3. 

Overall, the adsorption capacity of the β-CD-HDI and γ-CD-HDI polymers was similar, with the γ-CD-HDI polymer showing greater affinity towards guaiacol and 4-methylguaiacol, and the β-CD-HDI polymer showing a higher affinity for 4-ethylguaiacol and 4-ethylphenol. This difference in binding preference by β-CD and γ-CD was consistent with results from other experiments performed in the current study (outlined below). Among the four volatile phenols tested, it was obvious that 4-ethylphenol was by far the most readily removed compound. The adsorption capacity of the CD polymers for each of the volatile phenols (at equilibrium) was around 20 µg/g, and the adsorption capacity increased with increasing volatile phenol concentration. The magnitude of this adsorption is quite low compared with other studies [31,33,34,37]. This was attributed to the range of concentrations used in the current study, which were very low by comparison. Other studies have demonstrated that CD polymers, including β-CD-HDI, could achieve much greater adsorption at higher concentrations of the adsorbate. For example, the citric acid crosslinked β-CD polymer used by Zhao and colleagues exhibited a 13.8 mg/g adsorption capacity for phenol, with an equilibrium concentration of around 400 mg/L and a capacity of 3.8 mg/g at an equilibrium concentration of 85.4 mg/L [31]. The epichlorohydrin and chitosan crosslinked β-CD polymers used by Li and co-workers, showed similar adsorption capacities for phenol, *p*-nitrophenol and *p*-chlorophenol at equilibrium concentrations above 200 mg/L [34]; while Romo evaluated the β-CD-HDI polymer at higher levels of phenol and reported adsorption capacity of around 15 (mol_phenol_/mol_CD_) at an equilibrium concentration of 0.94 g/L [33]. In the current study, the starting and equilibrium concentrations of volatile phenols were no more than 1 mg/L, which are more reflective of concentrations observed in wine than concentrations reported in studies targeting pollutant removal from water. There is indeed limited literature reporting adsorption rates at these lower concentrations, so adsorption isotherm models were developed in the current study to predict the maximum adsorption capacity of the studied polymers. Several isotherm models have been developed to describe the adsorption of gaseous/liquid molecules onto an adsorbent surface. In the current study, Langmuir and Freundlich isotherm models were used to fit the data (Table 1).

The Langmuir isotherm assumes single-layer adsorption of adsorbate onto a homogenous surface with an identical cavity [34]. It is especially useful in describing adsorption at lower pressures (concentration of adsorbate), i.e., when the adsorption capacity curve (Figure 3) appears to be close to linear [38]. It can be expressed as:

q_e_ = 1/q_max_ K_L_ + C_e_/q_max_ (1)

where q_e_ is the adsorption capacity per unit weight of the polymer, q_max_ is the maximum adsorption capacity of the system, C_e_ is the residual concentration of volatile phenols at equilibrium, and K_L_ is the Langmuir isotherm constant. The equation can be re-arranged to give:

C_e_/q_e_ = 1/q_max_*C_e_ + 1/K_L_q_max_ (2)

It is obvious that if C_e_/q_e_ is plotted against C_e_, values of K_L_ and q_m_ can be calculated from the slope and intercept of the regression. In the current study, a Langmuir model fitted the data well with good linearity. The calculated values of K_L_ and q_m_ are shown in Table 1. The modelled maximum adsorption capacity (q_m_) for β-CD-HDI and γ-CD-HDI binding guaiacol, 4-methylguaiacol, 4-ethylguaiacol and 4-ethylphenol is between 19.6 mg/g (for γ-CD-HDI adsorbing guaiacol) and 25.1 mg/g (for β-CD-HDI adsorbing 4-ethylphenol), which falls within the ranges reported previously [31,34,36].

The Freundlich isotherm can be regarded as a special case of the Langmuir isotherm at intermediate adsorbate concentrations, where the adsorption capacity curve starts to plateau [39], and can be expressed as:

log q_e_ = log K_F_ + 1/n log C_e_ (3)

where K_F_ is the maximum adsorption capacity of the system, and 1/n is a natural value normally smaller than 1, which describes the extent of curving of the plotted log q_e_ against log C_e_, which relates to the ‘adsorption intensity’ or mobility of adsorbate at the adsorbent surface. The Freundlich isotherm fitted the data well in the current study. The K_F_ and 1/n for the Freundlich isotherm are also shown in Table 1. The K_F_ value is similar to the q_m_ predicted by the Langmuir isotherm, with the value ranging from 22.4 mg/g (for β-CD-HDI adsorbing 4-ethylguaiacol) to 33.1 mg/g (for β-CD-HDI adsorbing 4-ethylphenol). The 1/n value suggests homogeneity of the adsorption process across the experimental concentrations when it is close to 1, whereas a value close to 0 suggests heterogeneity. In the present study, the 1/n value ranged from 0.347 (for γ-CD-HDI adsorbing 4-ethylphenol) to 0.762 (for γ-CD-HDI adsorbing 4-methylguaiacol).

### 2.3. Batch Adsorption of Volatile Phenols by CD Polymers in Wine and Comparison with CD Addition

**Figure 4:**
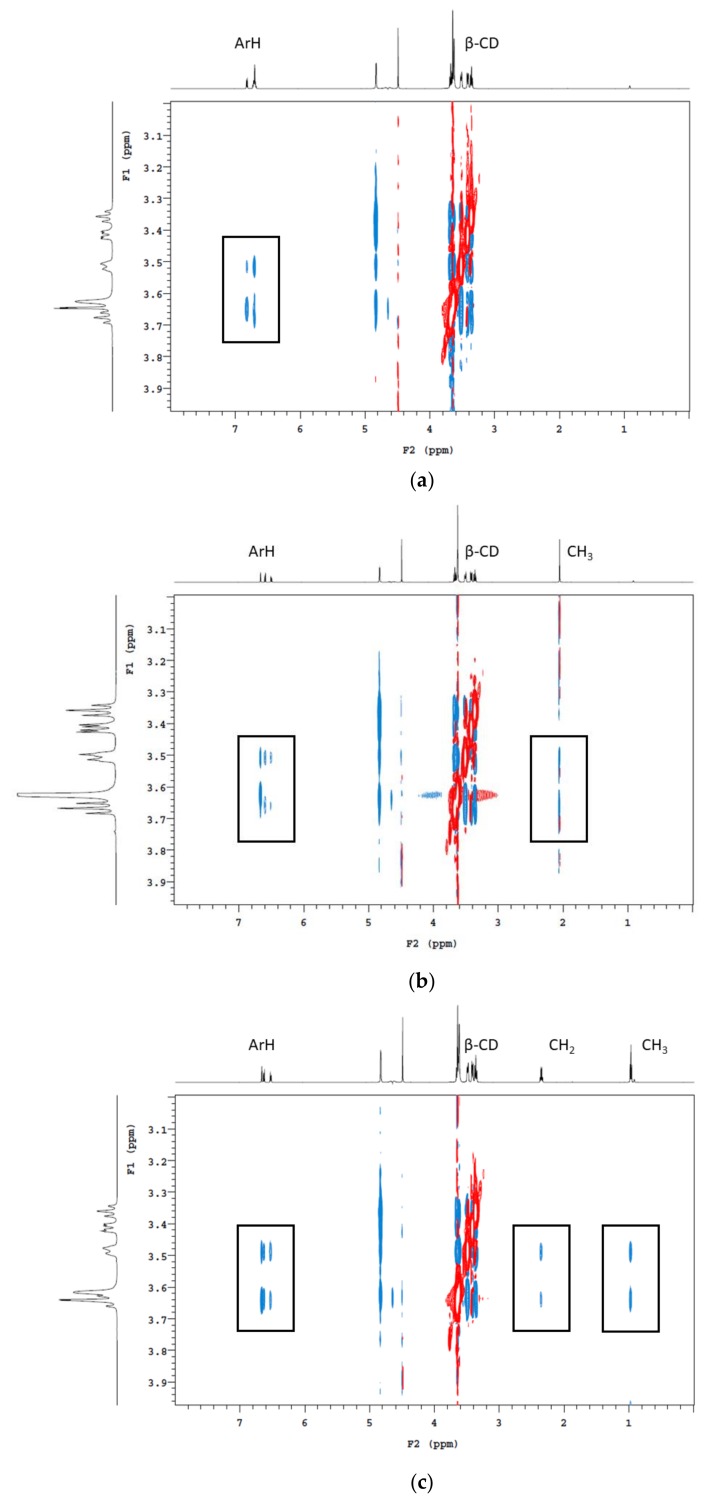
^1^H 2D ROESY NMR (600 MHz, pD 3.5 and 25 °C) spectrum of deuterated model wine containing β-CD and (**a**) guaiacol, (**b**) 4-methylguaiacol and (**c**) 4-ethylguaiacol. Rectangles indicate cross-peaks arising from nuclear Overhauser effect (NOE) interactions between the H3, H5, and H6 protons of the CD cavity and the alkyl and/or aromatic protons of the volatile phenols.

Three concentrations of the CD polymers were used in batch adsorption tests, with the percentage of volatile phenols removed shown in Table 2. Not surprisingly, with increasing amounts of polymer addition, the amount of volatile phenol being adsorbed also increased. Following the addition of β-CD-HDI and γ-CD-HDI polymers (5% *w*/*v*), the residual concentrations of guaiacol, 4-methylguaiacol, 4-ethylguaiacol and 4-ethylphenol were 55% and 54%, 55% and 51%, 43% and 46%, and 23% and 37%, respectively. When comparing these adsorption results with CD addition, it is worth mentioning that the concentrations of CD added were close to the limit of solubility (according to the supplier), which is a limiting factor for CD functionality. Nevertheless, the reduction in volatile phenol levels following addition of CDs was positively correlated with CD concentration, with 23 and 34% removal of guaiacol, 25 and 37% removal of 4-methylguaiacol, 42 and 43% removal of 4-ethylguaiacol, and 68 and 52% removal of 4-ethylphenol following addition of β-CD and γ-CD (at 20 g/L), respectively (Table 3). 4-Ethylphenol was consistently the most readily removed volatile phenol. The ranking of volatile phenols by the efficiency of removal (irrespective of polymer type) was: 4-ethylphenol > 4-ethylguaiacol > 4-methylguaiacol > guaiacol. It has been established that hydrophobicity and size are among the most influential factors in determining the extent of encapsulation of a guest molecule by CD [23,27]. The log P-value is the logarithm of a compound’s partition coefficient, and it is often used to describe the hydrophobicity of a compound, with higher log P values correlating with higher hydrophobicity. As expected, 4-ethylphenol has the highest hydrophobicity of the four volatile phenols studied, followed by 4-ethylguaiacol, with guaiacol being the lowest. When comparing the guaiacol-based volatile phenols, it is clear 4-methylguaiacol and 4-ethylguaiacol have a greater tendency to be encapsulated within the CD cavity due to the higher hydrophobicity granted by the alkyl group. This was apparent by 2D nuclear magnetic resonance (NMR) analysis (Figure 4). The cross-peaks indicate the close spatial correlation between protons. Spectra were cropped to specifically show interactions between the aromatic and alkyl protons of the volatile phenols with the CD cavity. It is obvious that the methyl and ethyl groups facilitate the insertion of 4-methylguaiacol and 4-ethylguaiacol into the CD cavity. 

Polymerisation appeared to enhance the binding capability of the CD cavity, with greater removal of volatile phenols achieved following addition of 5% *w*/*v* of CD polymer (Table 2), compared with addition of 20 g/L of the corresponding CD (Table 3); in the current study, the relative proportion of CD within 5% *w*/*v* of the CD polymer is approximately 20 g/L, taking molar ratios into account. The observed differences in binding affinity likely reflect changes in host-guest inclusion interactions due to modification of the polarity or reactivity of polymerised CD molecules and/or the orientation of encapsulated volatile phenols, as a consequence of cross-linking with HDI. 

### 2.4. Reusability Test 

**Figure 5:**
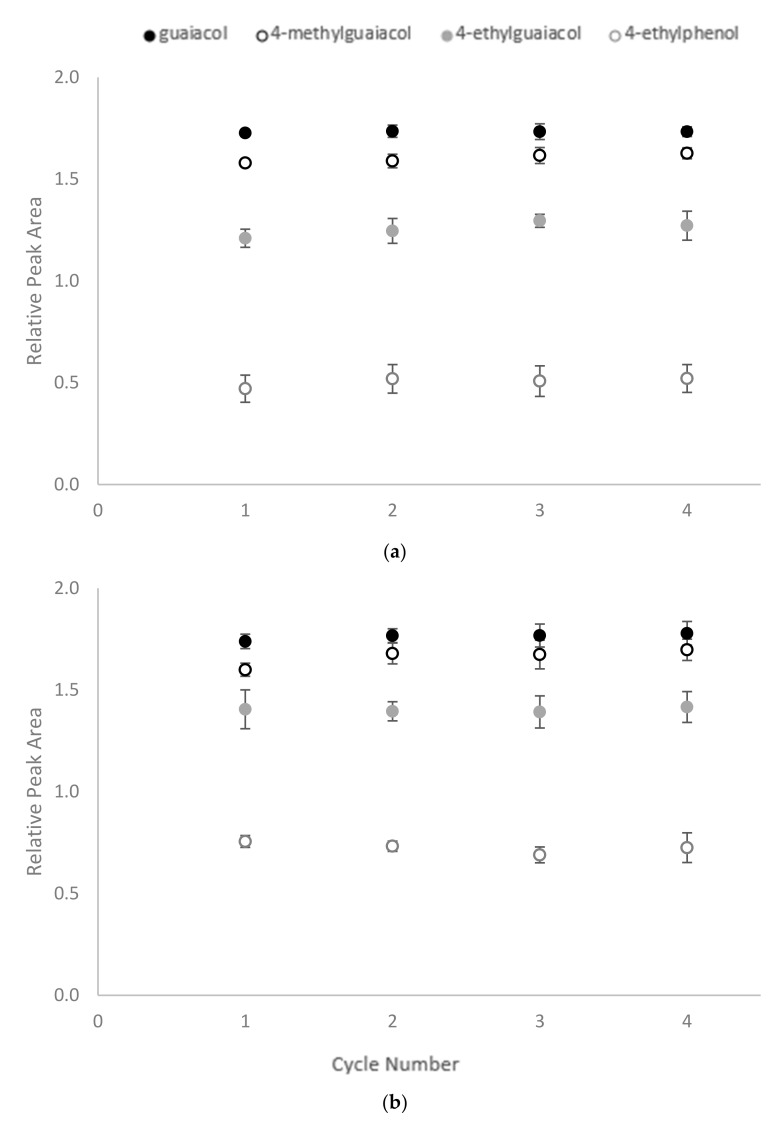
Reusability of CD-HDI polymers for sequential binding of volatile phenols by (**a**) β-CD-HDI and (**b**) γ-CD-HDI polymers, expressed as relative peak area. Values are means of three replicates ± standard error (but some standard errors are obscured by symbols).

Following treatment, the CD polymers were recovered and washed with methanol, before being added to fresh wine samples to determine their reusability. However, no significant differences were found between the RPA for residual volatile phenols across four cycles of adsorption (Figure 5). As mentioned above, the mechanism of CD encapsulation is mainly based on non-covalent bond hydrophobic interactions. The process can, therefore, be reversed by placing the CD polymers in a more hydrophobic environment, enabling them to be regenerated for repeated use.

## 3. Materials and Methods

### 3.1. Chemicals

Analytical grade volatile phenols (guaiacol, 4-methylguaiacol, 4-ethylguaiacol and 4-ethylphenol), hexamethylene diisocyanate (HDI), *N,N*-dimethylformamide (DMF), dibutyltin dilaurate and deuterated NMR solvents (*d*_6_-ethanol, D_2_O and DCl) were purchased from Sigma-Aldrich (Castle Hill, NSW, Australia). Analytical grade chloroform was purchased from Chem-Supply (Adelaide, SA, Australia) for polymer precipitation. The deuterium-labelled normalising standard, *d*_3_-4-methylguaiacol, was purchased from CDN Isotopes (Pointe-Claire, Quebec, Canada). Food grade (>98% purity) β-CD and γ-CD were sourced from the IMCD Group (Adelaide, SA, Australia). Stock solutions of volatile phenols and deuterated standards were made in pure ethanol (Thermo Fisher Scientific, Waltham, MA, USA) and stored at –20 °C. Working solutions were prepared in model wine containing 5 g/L of tartaric acid (Thermo Fisher Scientific) and 12% *v/v* ethanol, and were stored at –4 °C. A commercial red wine (a 2017 Barossa Valley Cabernet Sauvignon) was used for the adsorption study.

### 3.2. Preparation of HDI Crosslinked CD Polymers (CD-HDI)

CD-HDI polymers were prepared according to the method developed by Yamasaki and co-workers [30]. In the previously published method, polymerisation was achieved with a range of molar ratios of HDI to CD, due to the number of hydroxyl groups available for crosslinking within the CD structure. In the current study, the HDI:CD ratio was 4:1. To yield 5 g of polymer, 1.8 mM of β-CD or γ-CD was dissolved in 15 mL of DMF under a nitrogen atmosphere, with stirring. Two drops of dibutyltin dilaurate initiator were added to the mixture, before 7.2 mM of HDI in DMF (5 mL) was added. The crude product was heated in an oil bath at 70 °C under nitrogen. After 24 h, the resulting gel-like mixture was transferred into 50 mL of chloroform and stirred for 12 h to facilitate precipitation. The suspension was filtered and washed repeatedly, with around 10 L of de-ionised water. The polymer was then dried at 60 °C for 6 h before being ground in a multi-directional planetary QXQM-1 ball mill (Tencan, Changsha, China). The ground polymer was passed through a 150 µm sieve prior to its subsequent use. 

### 3.3. Adsorption Experiments with CD Polymers 

Kinetic adsorption tests were carried out (in triplicate) by adding 1% *w/v* polymer to model wine (10 mL) spiked with 1 mg/L of each of the volatile phenols (guaiacol, 4-methylguaiacol, 4-ethylguaiacol and 4-ethylphenol). The sample was agitated at 120 rpm at 25 °C for 2 h, during which time aliquots (1 mL) were periodically withdrawn (i.e., at 5, 20, 40, 80 and 120 min after the start of mixing) and centrifuged (2000 g for 5 min). The resulting supernatant was then analyzed by HS-SPME GC-MS (using established methodology [40]; with a Supelco DVB/CAR/PDMS fiber (Sigma Aldrich, Castle Hill, NSW) to determine volatile phenol concentrations. 

Since no significant difference was observed between RPAs for volatile phenols at 5 vs. 120 min after addition of CD-HDI polymers (i.e., adsorption equilibrium was achieved rapidly), subsequent analyses were conducted within 2 hours of incubation. To study the equilibrium adsorption capacity of the adsorbent in model wine, 2% *w/v* of polymer was added to 10 mL of model wine, containing various concentrations of guaiacol, 4-methylguaiacol, 4-ethylguaiacol or 4-ethylphenol. The concentration of volatile phenols ranged from 0.05 to 1.0 mg/L. Controls were prepared without the addition of polymer. The equilibrium adsorption capacity of the polymer is expressed as q_e_ (mg/g):

q_e_ = (C_0_ − C_e_)*V/W (4)

where C_0_ is the starting concentration of volatile phenols, C_e_ is the concentration of volatile phenols remaining at equilibrium, V is the sample volume, and W is the weight of the polymer. The concentration of volatile phenols remaining in the model wine was determined by HS-SPME GC-MS (again using established methodology [40]). 

To evaluate volatile phenol adsorption in a real wine environment, a batch experiment was conducted (in triplicate) to compare the adsorption efficiency (expressed as a percentage of the RPA) of the controls) using various amounts of CD-HDI polymers added to spiked red wine samples. The amount of CD-HDI polymer was 1%, 2% and 5% *w/v.* The reusability of the polymers was also tested. Pre-exposed polymers were collected from the wine samples and soaked in methanol at room temperature for 24 h with 120 rpm agitation, then filtered and dried for reuse. The regenerated polymers were subjected to five rounds of batch adsorption to test their regeneration efficiency. 

### 3.4. Binding Experiments Comparing CDs and CD Polymers

The two functional CDs, β-CD and γ-CD (in their monomer forms), were also evaluated in the current study to enable a comparison to be made with the performance of the CD polymers. CDs (20 g/L) were added to red wine samples spiked with 1 mg/L of volatile phenols. Samples were then incubated at 35 °C with 120 rpm agitation for 20 min, prior to analysis using the four-phase HS-SPME GC-MS method [35]. Controls (without any CD addition) were also prepared and analyzed (in triplicate). 

### 3.5. Four-Phase HS-SPME GC-MS Analysis

A recently developed four-phase HS-SPME GC-MS method was used (with minor modification) to quantify residual volatile phenol levels [35]. An ampoule containing 0.2 mL of model wine solution was spiked with the isotopically labelled standard (*d*_3_-4-methylguaiacol, at 2 mg/L) and inserted into a 20 mL headspace sampling vial (Sigma Aldrich, Castle Hill, NSW, Australia), containing 0.2 mL of the sample to be analyzed. The vial was then incubated for 5 min at 35 °C. The fiber extraction of headspace aroma compounds occurred over 15 min without agitation. Samples were then analyzed using an Agilent 6890 GC-MS system coupled to a 5973 mass selective detector (Santa Clara, CA, USA), fitted with a Gerstel MPS autosampler (Mülheim, Germany). The column used for GC separation was a 60 m DB-Wax column with 0.25 mm internal diameter and 0.25 µm film thickness (Agilent J&W, Folsom, CA, USA). Helium (BOC Gas, Adelaide, SA, Australia) was used as the carrier gas at a constant flow of 1.5 mL/min. The inlet temperature was set at 240 °C. The oven temperature started at 40 °C for 5 min, then increased to 250 °C at 3 °C/min and was held at 250 °C for 5 min, to give a total run time of 80 min. Selected Ion Monitoring (SIM) mode was used to record the mass spectra of target ions. The ions monitored in SIM mode were: *m*/*z* 109, *124* for guaiacol; *m*/*z* 123, *138* for 4-methylguaiacol; *m*/*z* 126, *141* for *d_3_*-4-methylguaiacol; *m*/*z* 122, 137, *152* for 4-ethylguaiacol; and *m*/*z* 77, *122* for 4-ethylphenol; with italicised ions used for quantitation. Volatile phenol concentrations are reported as relative peak areas (RPA), i.e., as the ratio of the peak area of the analyte relative to the peak area of the normalising standard (*d*_3_-4-methylguaiacol).

### 3.6. Nuclear Magnetic Resonance Analysis

Encapsulation of volatile phenols by CDs was investigated by two-dimensional NMR rotating frame Overhauser effect spectroscopy (^1^H 2D ROESY), using an Agilent DD2 600 MHz spectrometer fitted with a cryoprobe (Agilent Technologies, Santa Clara, CA, USA), operating at 600 MHz with a mixing time of 300 ms. Samples were prepared by adding guaiacol, 4-methylguaiacol or 4-ethylguaiacol to deuterated model wine (12% d_5_-ethanol in D_2_O, pD adjusted to 3.5 by dropwise addition of DCl) containing β-CD, as previously reported for 4-ethylphenol [35].

### 3.7. Data Analysis

Data are presented as mean values of three replicates ± standard error. One-way analysis of variance (ANOVA) was conducted to determine differences between sample means, with a T-test at *p* = 0.05, using XLSTAT software (version 2015.3, Addinsoft, Paris, France).

## 4. Conclusions

The two CD polymers tested in the current study, β-CD-HDI and γ-CD-HDI, were found to be capable of removing volatile phenols from both model wine and red wine, at volatile phenol concentrations that reflect heavily tainted wines. Considering the practical use of CDs for off-odor removal in wine, the use of polymers affords several advantages over CDs; not only does the solubility of CDs limit their functionality, but the addition of CD may negatively impact overall wine quality and needs to be investigated further. In comparison, polymerised CDs could be used as a fining agents (pending classification as permitted winemaking additives), with polymerisation appearing to enhance the binding capability of the CD cavity. The use of CD polymers as innovative materials for mitigating off-odors in wine has therefore been demonstrated; the sensory impact of CD polymer addition to tainted wines, together with the potential for desirable wine constituents to be encapsulated by CD polymers, are the subject of ongoing research. 

## Figures and Tables

**Table 1:** Langmuir and Freundlich isotherms for the adsorption of volatile phenols by β-CD-HDI and γ-CD-HDI in model wine at 25 °C.

		Langmuir Adsorption Isotherm			Freundlich Adsorption Isotherm		
		q_m_ (mg/g)	K_L_ (L/mg)	r	K_f_	1/n	r
guaiacol	β-CD-HDI	22.3	3.0	0.989	21.7	0.67	0.992
	γ-CD-HDI	19.6	5.8	0.992	22.3	0.56	0.991
4-methylguaiacol	β-CD-HDI	20.1	4.9	0.990	21.5	0.56	0.987
	γ-CD-HDI	21.2	5.8	0.988	23.5	0.54	0.995
4-ethylguaiacol	β-CD-HDI	20.0	10.4	0.995	23.9	0.44	0.968
	γ-CD-HDI	20.5	7.7	0.997	24.2	0.50	0.959
4-ethylphenol	β-CD-HDI	25.1	8.1	0.986	34.0	0.56	0.957
	γ-CD-HDI	20.6	23.1	0.976	25.9	0.35	0.985

q_m_ = the maximum adsorption capacity; K_L_ = the Langmuir isotherm constant; r = the correlation coefficient; K_f_ = the Freundlich constant; and n = the constant related to the adsorption intensity.

**Table 2:** Residual volatile phenols levels (as relative peak area) following addition of β-CD-HDI and γ-CD-HDI to red wine spiked with volatile phenols.

		Guaiacol		4-methylguaiacol		4-ethylguaiacol		4-ethylphenol	
control		2.34 a ± 0.03		2.18 a ± 0.04		1.89 a ± 0.03		0.97 a ± 0.03	
	1% *w/v*	2.07 b ± 0.03	89%	1.96 a ± 0.01	90%	1.63 b ± 0.01	86%	0.81 b ± 0.01	84%
β-CD-HDI	2% *w/v*	1.69 c ± 0.05	72%	1.57 b ± 0.03	72%	1.25 c ± 0.02	66%	0.55 c ± 0.01	57%
	5% *w/v*	1.29 d ± 0.05	55%	1.17 c ± 0.05	55%	0.82 d ± 0.02	43%	0.23 d ± 0.01	23%
	1% *w/v*	1.97 b ± 0.06	84%	1.90 a ± 0.05	87%	1.63 b ± 0.05	86%	0.83 ab ± 0.04	86%
γ-CD-HDI	2% *w/v*	1.61 c ± 0.10	69%	1.50 b ± 0.10	69%	1.31 c ± 0.00	69%	0.64 c ± 0.00	66%
	5% *w/v*	1.27 d ± 0.04	54%	1.11 c ± 0.10	51%	0.88 d ± 0.08	46%	0.36 d ± 0.06	37%

Values are means of three replicates ± standard error (and percentage of control). Values followed by different letters are significantly different (one-way ANOVA, *p* = 0.05).

**Table 3:** Residual volatile phenols levels (as relative peak area) following addition of β-CD and γ-CD to red wine spiked with volatile phenols.

		Guaiacol		4-methylguaiacol		4-ethylguaiacol		4-ethylphenol	
control		2.43 a ± 0.06		2.30 a ± 0.09		1.99 a ± 0.08		1.04 a ± 0.05	
	5 g/L	2.06 bc ± 0.02	85%	1.88 b ± 0.07	82%	1.62 b ± 0.07	81%	0.79 b ± 0.01	76%
β-CD	10 g/L	1.96 bc ± 0.02	81%	1.80 b ± 0.07	78%	1.38 bc ± 0.07	69%	0.49 c ± 0.01	47%
	20 g/L	1.87 d ± 0.01	77%	1.73 bc ± 0.02	75%	1.15 c ± 0.03	58%	0.33 c ± 0.00	32%
	5 g/L	2.19 b ± 0.06	90%	1.97 ab ± 0.06	85%	1.64 b ± 0.05	82%	0.89 ab ± 0.03	86%
γ-CD	10 g/L	1.83 cd ± 0.08	75%	1.68 bc ± 0.10	73%	1.41 bc ± 0.09	71%	0.76 b ± 0.07	73%
	20 g/L	1.61 d ± 0.05	66%	1.45 c ± 0.10	63%	1.13 c ± 0.04	57%	0.49 c ± 0.01	48%

Values are means of three replicates ± standard error (and percentage of control). Values followed by different letters are significantly different (one-way ANOVA, *p* = 0.05).

## Supplementary Materials

The following are available online at https://www.mdpi.com/1420-3049/25/4/910/s1, Figure S1: Chemical structures of guaiacol, 4-methylguaiacol, 4-ethylguaiacol and 4-ethylphenol.
