# Supplementary material for: Removal of Volatile Phenols From Wine Using Crosslinked Cyclodextrin Polymers
Source: Molecules. 2020 Feb 18;25(4):910. doi: 10.3390/molecules25040910 (PMC7070489; doi:10.3390/molecules25040910)
Supplement: Supplementary file 1 [file molecules-25-00910-s001.pdf]

Supplementary Materials

Removal of volatile phenols from wine using crosslinked cyclodextrin polymers

Chao Dang [1,2], Vladimir Jiranek [1,2], Dennis K. Taylor [1,2] and Kerry L. Wilkinson [1,2,*]

[1] The University of Adelaide, School of Agriculture, Food and Wine, PMB 1, Glen Osmond, SA, 5064, Australia; chao.dang@adelaide.edu.au (C.D.), vladimir.jiranek@adelaide.edu.au (V.J.), dennis.taylor@adelaide.edu.au (D.K.T.)

[2] The Australian Research Council Training Centre for Innovative Wine Production, PMB 1, Glen Osmond, SA, 5064, Australia

* Correspondence: kerry.wilkinson@adelaide.edu.au (K.L.W.); Tel.: +61-8-8313-7360

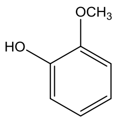

guaiacol

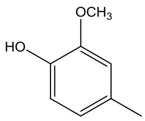

4-methylguaiacol

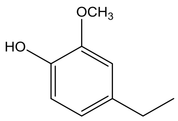

4-ethylguaiacol

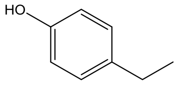

4-ethylphenol

Figure S1. Chemical structures of guaiacol, 4-methylguaiacol, 4-ethylguaiacol and 4-ethylphenol.
